# Drugging Pockets at the Surface of c‑MYC Oncoproteins Revealed by Well-Tempered Metadynamics

**DOI:** 10.1021/acs.jcim.6c02197

**Published:** 2026-10-22

**Authors:** Yanhong Ge, Huixia Lu, Jordi Marti

**Affiliations:** † Department of Physics, Universitat Politècnica de Catalunya-Barcelona Tech (UPC), Campus Nord UPC, Barcelona 08034, Spain; ‡ Institut de Ciència de Materials de Barcelona (ICMAB-CSIC), Carrer dels Til·lers, Cerdanyola del Vallès 08193, Spain

## Abstract

The MYC oncogene encodes a master transcription factor essential for regulating cellular homeostasis, growth, and metabolism. However, its deregulation is involved in up to 70% of human cancers, where it drives uncontrolled cellular proliferation. Structurally, MYC is characterized by an N-terminal transactivation domain and a C-terminal bHLHLZ domain critical for DNA binding and dimerization with MAX. Despite its clinical significance, MYC has remained historically “undruggable” due to its intrinsically disordered structure and the absence of traditional binding pockets. In this study, we combined all-atom Molecular Dynamics and well-tempered metadynamics simulations to map the conformational landscape of a MYC motif in aqueous ionic solution. The selected energy-minimized structures exhibit stable conformations and drugging pockets, providing structural foundations for targetting c-MYC. Furthermore, both the electrostatic potential and docking results for the obtained structure and the MYC inhibitor’s 10074-G5 demonstrated excellent fit, highlighting its potential as a drug target. This inhibitor, introduced in several previously unpublished drugging pockets, maintained a steady binding to MYC over unbiased trajectories lasting 1 μs. These findings provide a previously uncharacterized structural foundation for the design of innovative therapeutic interventions, such as small-molecule inhibitors or peptides aimed at disrupting oncogenic MYC activity.

## Graphical abstract

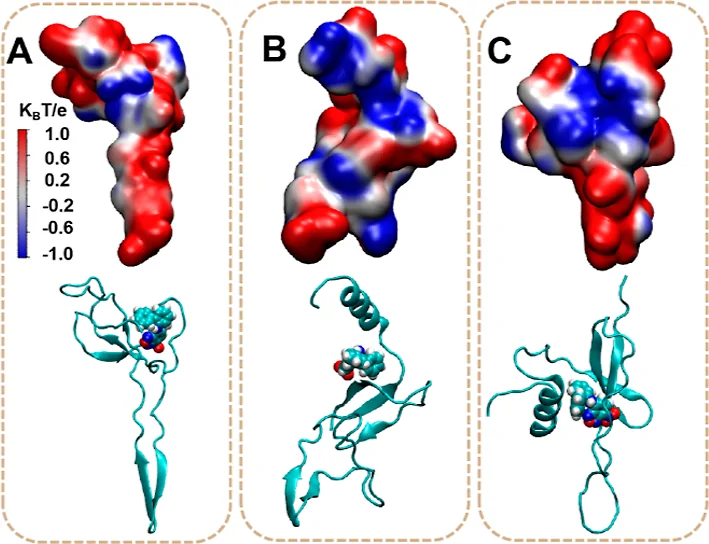

## Introduction

MYC oncogenes encode a transcription factor that plays a crucial role in regulating numerous cellular processes, including growth, differentiation, metabolism, apoptosis, and DNA repair.^1–4^ MYC is vital for normal cellular development, but its overexpression or mutation can drive tumorigenesis. MYC is highly conserved between species,^5^ reflecting its essential role in maintaining cellular homeostasis and cell cycle progression. First identified within the avian retrovirus MC29,^6,7^ the c-MYC gene was one of the first oncogenes to be recognized, and its evolutionary conservation further underscores its biological importance. MYC consist of three key regions: a transactivation domain at the N-terminus, a central region, and the basic helix–loop–helix leucine zipper (bHLHLZ) domain at the C-terminus. The bHLHLZ domain is characteristic of a superfamily of transcription regulators that mediate cellular responses to extracellular and intracellular signals by modulating gene expression.^8^ This domain enables MYC to bind to DNA through E-boxes, specific sequences that facilitate its interaction with various cofactors, particularly the MAX protein.^9,10^ In many cancers, MYC is overexpressed, disrupting the balance between MYC activation and repression. This imbalance favors MYC–MAX complexes, which promote uncontrolled cell growth and inhibit differentiation. Although MYC was once thought to require MAX for both activation and repression, recent studies have shown that MYC retains significant biological activity even in the absence of MAX,^11^ further emphasizing the importance of the MYC monomer as a target. MYC expression is deregulated in up to 70% of human cancers,^1^ including Burkitt lymphoma, colorectal cancer, melanoma, prostate cancer, breast cancer (via translocation or amplification of c-MYC) and neuroblastoma (via amplification of n-MYC), among others.^7,12^

Despite its critical role in oncogenesis, targeting MYC directly remains challenging due to its lack of easily druggable sites, since MYC is an intrinsically disordered protein (IDP) that does not present obvious binding pockets for small molecule inhibitors.^13–16^ As a result, direct strategies to inhibit MYC function have focused on indirect approaches,^1,17,18^ such as disrupting the dimerization of MYC–MAX, preventing MYC–DNA binding, or interfering with MYC target gene expression.^19^ As one particularly relevant approach to prevent the formation of MYC–MAX complexes, we can mention the finding of a group of seven inhibitors by Yin et al.^20^ including the inhibitor 10074-G5 among them (*N*-([1,1′-biphenyl]-2-yl)-7-nitrobenzo­[*c*]­oxadiazol-4-amine),^21,22^ as MYC small-molecules impeding the MYC–MAX complex formation, consequently attenuating the proliferative activity of tumor cells.^20,21^ In particular, it has been found that 10074-G5 binding sites to c-MYC are residues 366–375 of the bHLHZip domain.^23^ However, characterizing the conformational landscape of c-MYC is a highly complex task due to its intrinsically disordered nature. Extensive biophysical and simulation studies^24–27^ have established that small-molecules target c-MYC through a diffuse, heterogeneous mechanism, often described as a “ligand cloud” interacting with a “protein cloud”.^28^ Rather than trapping a single rigid conformation, inhibitors like 10074-G5 disrupt MYC–MAX dimerization by interacting with an ensemble of transiently populated states. In this context, exploring the free energy surface via advanced sampling techniques like well-tempered metadynamics (WTM) allows us to identify the underlying metastable basins and local pockets that are transiently sampled by the protein, providing a structural basis for understanding these dynamic interactions. More recently, the use of Markov state models has been considered to understand the bonding of small-molecules such as 10058-F4, producing better binding energy estimates than alchemical free energy calculations.^26^ Recently, an “ensemble-docking” perspective has arisen to extend the search of particular docking sites to generate conformational ensembles of ligand binding modes.^27^ An alternative different way to prevent dimerization is to use modified proteins. For example, an innovative approach involves Omomyc,^29^ a mutant of MYC engineered to disrupt MYC dimerization. Omomyc contains a modified bHLHLZ domain that allows it to homodimerize and heterodimerize with MYC and MAX, disrupting the normal MYC function. Early clinical trials with Omomyc have shown promising results, indicating that it could be a potential therapeutic option.

In this study, we leverage Molecular Dynamics (MD) simulations at the all-atom level, which provide high temporal resolution and precise force fields to model the behavior of MYC and its interactions with other proteins and drugs. Interestingly, MD can model hydrogens and compute absorption spectra at the classical^30–32^ or quantum levels^33^ although in biological simulations a classical rigid model for water (TIP3P) is usually taken. On the one hand, by analyzing the conformational dynamics of MYC we aim to uncover potential druggable sites and better understand the mechanisms of MYC dimerization and DNA binding. Furthermore, the description of the stable states on the free energy hypersurface of isolated MYC in solution, which can be accurately reproduced using tools such as WTM simulations, is a key information to combine with MD data and help the location of pockets or regions at the MYC surface to be targeted, paving the way for future therapeutic interventions based on the design of new drugs. In the present work, structural and free energy data have been combined in order to gather key information on: (1) stable dynamical structures of MYC in aqueous ionic solution, obtained from both MD and WTM, and (2) drugging pockets located on the surface of MYC, which can potentially be targeted by a variety of compounds, some of them being currently designed and evaluated by *in silico* techniques. To validate the stability of the drug pocket, we docked the lowest-energy conformation identified in WTM with a MYC inhibitor (10074-G5) and simulated the resulting complex, thereby providing a structural basis for the design of brand new small-molecule inhibitors.

## Methods

### System Preparation and Setups

In the present work, we conducted MD and WTM simulations of a motif species c-MYC in sodium chloride aqueous solution, with the sequence^34^ expressed by 82 amino acids: NVKRRTHNVLERQRRNELKRSFFALRDQIPELENNEKAPKVVILKKATAYILSVQAEEQKLISEEDLLRKRREQLKHKLEQL. Three crystal structures (CS) of the selected MYC motif were extracted from the PDB files “1NKP”, “8OTS” and “6G6K” from the RCSB Protein Data Bank.^35,36^ Every simulated system contained one motif MYC protein fully solvated by TIP3P water molecules^37^ in NaCl at the human body concentration (0.15 M). After constructing the corresponding initial setups, we performed one independent classical MD simulation of 3 μs for each structure. In addition, a replica simulation of 2 μs was conducted for each system as a control, resulting in a total of six MD trajectories, with an aggregate simulation time of 15 μs. We selected the residue segment 354–432 from 8OTS. In order to standardize the residue lengths of the three initial structures, we manually added the C, N and O atoms for the terminal residues 353, 433 and 434, and used CHARMM-GUI solution builder^38–40^ to complete the remaining hydrogen atoms. The resulting complete structures retained the original helical structure at both ends.

The total number of particles in these six MD systems ranged between 223,305 and 227,872 atoms and the corresponding sizes of the simulations boxes were between 133.8 × 133.6 × 133.4 Å^3^ and 135.5 × 134.9 × 134.9 Å^3^, in order to get the MYC motif fully hydrated and located far enough from its replica images. All inputs were generated by means of CHARMM-GUI, assuming the CHARMM36m force field.^41^ All bonds involving hydrogens were set to fixed lengths, allowing fluctuations of bond distances and angles for the remaining atoms. The MYC proteins in their solvated water box were energy minimized and well equilibrated (*NVT* ensemble) before generating MD (*NPT* ensemble) and WTM simulations. MD trajectories were used to feed the initial configurations of the WTM runs. Once we have systematically verified the physical equivalence of the three structures, we have obtained averaged properties in all cases.

The GROMACS/2023 package^42^ was employed for the system minimization as well as for the equilibration and production steps of the MD simulations. The system was minimized with steepest descents and a conjugate-gradient step every 10 steps until convergence to *F*_max_ ≤ 1000 kJ/mol. After energy minimization, a time step of 2 fs was used in all equilibration simulations and the particle mesh Ewald method with Coulomb radius of 1.2 nm was employed to compute long-ranged electrostatic interactions. The cutoff for Lennard-Jones interactions was set to 1.2 nm. The system was equilibrated for 1.25 μs with the Nosé–Hoover thermostat set at 310.15 K in the *NVT* ensemble, before switching to the *NPT* ensemble at pressure 1 atm to collect statistics. Pressure was controlled by means of the Nosé–Hoover barostat. Periodic boundary conditions (PBC) in the three directions of space were taken in all cases.

### Well-Tempered Metadynamics

A broad range of methodologies have been proposed to address the challenging task of computing free energy landscapes in multidimensional systems.^43–47^ These approaches aim to capture the intricate behavior of complex systems across multiple degrees of freedom. In the present study, we have utilized the WTM method, which has proven to be particularly effective in exploring free energy surfaces in systems with multiple reaction coordinates. This method has demonstrated significant success in tackling the free energy landscapes of a wide array of complex systems.^48–53^ Notably, WTM has shown remarkable efficacy when used to study model cell membrane systems, including those with attached small molecules and proteins.^54–56^ This makes it particularly well-suited to the investigation of biological processes at a molecular level, which has been a primary focus of recent work in our research group. The ability of WTM to handle the complexity of such systems, including their dynamic interactions and high-dimensional configuration spaces, positions it as a powerful tool for advancing our understanding of molecular mechanisms in various biological contexts.

Extensive WTM simulations considering different collective variables (CV) with total lengths between 7.8 and 10 μs were performed, and once two suitable CV were determined (see section “Collective Variables” for the choice of CV), we obtained the Gibbs free energy (hyper)­surface (FES), starting from the last configuration of one MD simulation (labeled as “replica 1”, see below), considering a Nosé–Hoover thermostat at 310.15 K and a Parrinello–Rahman barostat at 1 atm in the *NPT* ensemble. These WTM simulations were performed using PLUMED, through the joint GROMACS/2023-plumed-2.9.2 tool.^57,58^ As in the MD simulations, PBC in the three directions of space were also considered. The total number of atoms in the setups for the WTM simulations was of 39,489 with a box size of 75.0 × 75.0 × 75.0 Å^3^.

In WTM, a useful tool is the integration of CVs from a high-dimensional FES in order to reduce the dimensionality and favor the physical interpretation, when possible by comparison to experimental data. To do so, in the present work, 1D free energy profiles *F*(*s*_1_) (comparable to the experimental Δ*G*_bind_) can be obtained by means of eq 1 ^59,60^

1 $$F\left(s_{1}\right)=-\beta^{-1}\;\ln\left[\frac{\int e^{-\beta F\left(s_{1},s_{2}\right)}\;ds_{2}}{\int e^{-\beta F\left(s_{1},s_{2}\right)}\;ds_{1}\;ds_{2}}\right]$$

where *s*_1_ and *s*_2_ are the CV, β = 1/(*k*_B_ *T*), *k*_B_ is the Boltzmann constant and *T* is the absolute temperature. Here, the free energy *F*(*s*_1_) depends only of *s*_1_, whereas all possible contributions due to *s*_2_ have been integrated out and averaged.

### Collective Variables

The enhanced sampling method WTM applies a time-dependent biasing potential along a set of CV by adding a Gaussian additional bias potential to the total potential in order to overcome barriers larger than *k*_B_ *T*, with *k*_B_ being Boltzmann’s constant and *T* the temperature. In the method, sampling is performed on a selected number of degrees of freedom (*s*_*i*_), i.e. a chosen set of CV. For each degree of freedom, the biased potential *V*(*s*_*i*_, *t*), described in eq 2, is a dynamical function constructed as the sum of Gaussian functions

2 $$V\left(s_{i},t\right)=\sum_{k\tau <t}W\left(k\tau \right)\;\exp\left\{-\sum_{i=1}^{d}\frac{{\left(s_{i}-s_{i}\left(q\left(k\tau \right)\right)\right)}^{2}}{2\sigma_{i}^{2}}\right\}$$

where *k* is an integer, τ is the Gaussian deposition stride, *W*(*k*τ) is the height of the Gaussian, σ_*i*_ is the width of the Gaussian for the *i*-th CV and *d* is the number of CV. Further details are provided elsewhere.^61–63^ Unlike unbiased MD simulations, able to track the dynamical evolution of the system around equilibrium states (stable states), the biased potential can force the system to move around all possible states inside a particular range of the subspace of selected CV. We should point out here that determining and using three or more CVs in a WTM simulation is practically unsuitable from the computational point of view.^48^ The main reasons are (1) three or more CVs extend exponentially the required sampling, so when the system is large, as it is in the present case, reaching correct convergence requires simulation times or the order of hundreds of microseconds (the present work required about 8 μs); (2) Gaussian deposition becomes less efficient: since each Gaussian occupies a small volume of CV space, to cover the same space in high dimensions one needs many more Gaussians; (3) the exploration of additional CV space is usually irrelevant, since one has to cover a substantial range of each CV in order to locate the relevant free energy basins. Finally, the comparison to experimental data is often performed through projections of the full free energy multidimensional profile on a single variable.^59^

The usual choice is to consider one or two CVs.^64^ The use of two CVs produces a complete enough description of the FES and we have observed this is the optimal way to proceed.^65^ Later on, we will project the free energies onto one single coordinate, integrating out the contribution of the second CV. The values of the parameters taken for the WTM simulations^62^ are listed in Table 1.

**Table 1:** Two-dimensional WTM simulation parameters of aqueous c-MYC systems, where the CVs are *d*_1_ and *d*_2_

parameter	value
Gaussian width of *d*_1_ [nm]	0.082
Gaussian width of *d*_2_ [nm]	0.035
Gaussian hill [kJ/mol]	1.0
deposition stride [ps]	20
bias factor	10
simulation time [μs]	7.8

In this work, we have taken a specific approach similar to previous works^54,66,67^ where CVs were defined to obtain clear and well-defined free energy landscapes. After a detailed study of several distances and angular coordinates, the two CV selected to perform the 2D WTM calculations are shown in Figure 1. We have selected: (1) as CV1, the distance (*d*_1_) between the residue ASN907 within the α helix close to the C-terminal of MYC and the residue HIS976 within the second α helix, close to the N-terminal of MYC; and (2) as CV2 the distance (*d*_1_) between residues LEU951 and LEU966, both located at the central part of the second α helix of MYC, in order to study the potential torsions of this particular α helix.

**Figure 1:**
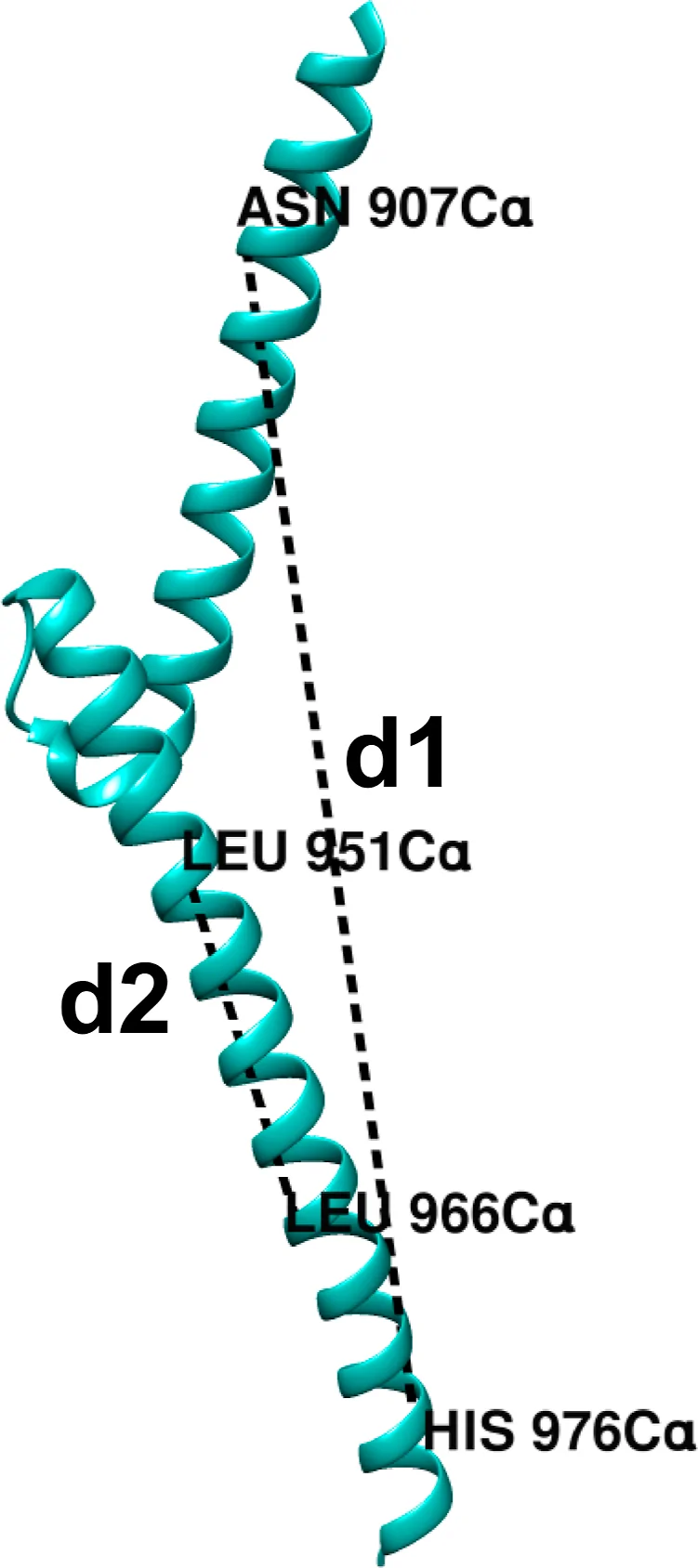
Schematic diagram of the atoms involved in the definition of CVs: (1) the distance (*d*_1_) between the ASN907 α carbon atom and the HIS976 α carbon atom and (2) the distance (*d*_2_) between the LEU951 α carbon atom and the LEU966 α carbon atom.

Specifically, *d*_1_ is a global variable, while *d*_2_ serves as a local variable for the long α helix region. Since clustering analysis indicates that the ends of the helices tend to uncoil and to avoid interference introduced by the highly flexible terminal residues and the intermediate loop, both *d*_1_ and *d*_2_ exclude the N-terminus (residues 900–906) and C-terminus (residues 977–981) and loop (residues 927–937), which lack a well-defined structure.

As a relevant remark, we should inform the reader about the importance of setting the correct parameters and specifications for the WTM simulations within the PLUMED project tool employed in the present work. To ensure physically meaningful distance-based CVs during metadynamics simulations, careful treatment of periodic boundary conditions is essential. In PLUMED, distances between atoms or molecular groups may be evaluated using the nearest periodic image, which can produce artificially short distances when the system spans across the simulation box. In the present work, this issue was resolved by combining the “WHOLEMOLECULES” directive with the “NOPBC” option in the CV’s definition. The “WHOLEMOLECULES” command reconstructs fragmented molecular coordinates across periodic boundaries prior to CV evaluation, while “NOPBC” ensures that the distance is computed using the actual molecular geometry rather than the minimum-image convention.

Figure 2 illustrates the importance of this treatment. In panel A, where “WHOLEMOLECULES” and “NOPBC” were properly applied, the trajectory-derived distance is in excellent agreement with the values reported in the “COLVAR” file (which stores all values of the 2 CVs during the simulation). This confirms that the CV accurately represents the physical separation between the selected groups. In contrast, panel B shows the result obtained without proper PBC handling, where commands “WHOLEMOLECULES” and “NOPBC” were not considered. Further details can be seen in the corresponding Github repository (https://github.com/yanhong77634/myc). In panel B, although the trajectory indicates a progressive increase in the intermolecular distance, the corresponding “COLVAR” values remain artificially low because PLUMED evaluates the distance to a periodic image of the molecule. These results emphasize that reconstruction of molecular continuity together with explicit disabling of periodic-image distance evaluation is critical for reliable enhanced-sampling simulations involving large conformational displacements or intermolecular separation processes.

**Figure 2:**
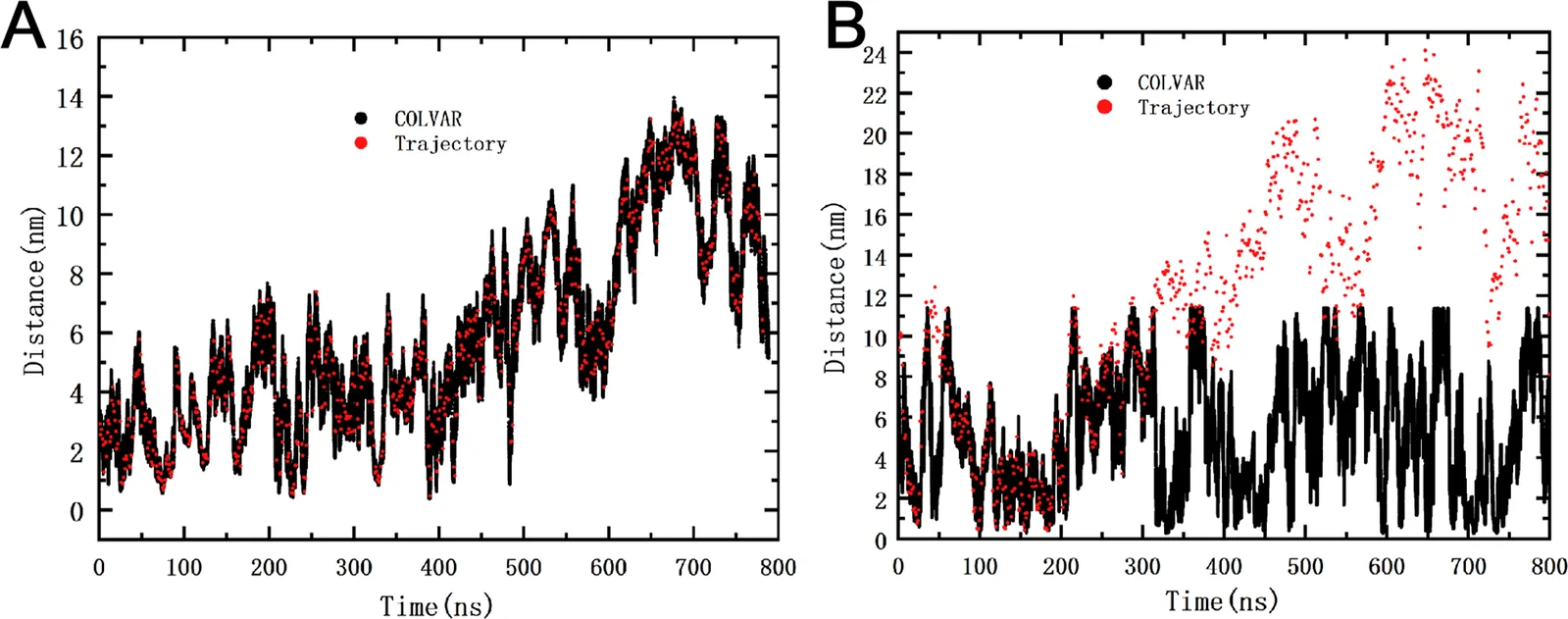
Comparison of values of CV *d*_1_, that corresponds to the distance between the ASN907 and the HIS976 alpha carbons of MYC in a MD trajectory (red dots) and the values in the “COLVAR” file calculated by PLUMED (full line) with the correctly configured PLUMED file (A) and using a wrong configuration (B).

### Data Analysis

#### Principal Component Analysis

Principal component analysis (PCA) is a statistical technique used to reduce the dimensionality of data while retaining as much variance as possible. It works by transforming the original variables into a new set of uncorrelated variables called principal components, which are ordered by the amount of variance they capture. The first few principal components often capture the majority of the data’s information, allowing for data compression and simplifying analysis. A comprehensive review of PCA’s applications and theory was published by Jolliffe.^68^ There are two principal components in our PCA study. The first component comprises the pairwise Euclidean distances between all *C*_α_ atoms, yielding a total of (164 × 163)/2 features. These distances describe the relative spatial variations among internal atoms throughout the protein, thereby capturing global conformational differences. The second component is the distance between the centers of mass of residue groups 900–981 and 200–281, providing a single feature that reflects the conformational changes of the second α helix (close to the N-terminal of MYC). Subsequently, this collective variable matrix is reduced to two principal components to facilitate visualization and clustering.

#### The R-Package

Metadynminer was used to analyze the WTM results, such as calculating the free energy surface, finding basins of minimal free energy and analyzing transition pathways between stable states (minimum free energy paths, MFEP) in order to describe the most probable transition routes between these states on the FES and to eventually locate the transition states (TS) of the system.^69^ The MFEP represent the reaction pathways with the lowest energy demand between two stable states.

The *neb* function of metadynminer also estimates the half-life of these states^70^ using the classical Eyring–Polanyi equation of chemical kinetics,^71^ with a transmission coefficient assumed to be 1. Hence, transition half-life is calculated from eq 3

3 $$t_{1/2}=\frac{\ln2}{k}$$

where *k* is derived from the Eyring–Polanyi equation. It represents how long a system remains in one stable state before transitioning across an activation barrier to another stable state. In other words, it is the time required for half of a starting population of molecules to cross from a stable state through an energy barrier into a new stable state. It is worth noting that these values should not be interpreted as physically meaningful transition times between the structurally distinct conformations shown in their corresponding free energy landscape. Instead, half-lives are consistent with the number of conformers sampled for each basin in the WTM trajectories. Shorter half-lives simply indicate faster kinetic transitions between states. Finally, the software VMD^72^ was used for visualization and analysis purposes.

## Results and Discussion

This section has been distributed into three main blocks. The first block is devoted to the construction of MD trajectories generated by starting from three different crystal structures obtained from PDB sources. The second one describes the two-dimensional free energy surface of MYC with full details, including the calculation of minimum free energy paths between stable states and the location of the most relevant transition states between stable states, together with its projection onto each CV. The third part is devoted to the characterization of the potentially targetable drugging pockets, revealed as stable basins located at the FES. We will close the “Results and Discussion” section with a study of the docking of the main stable structures with a prototype small-molecule inhibitor and the simulation of the resulting complexes to validate the stability of the drugging pocket.

### Stable Structures from Principal Component Analysis

As it has been detailed in section “Methods”, MD was conducted on six parallel runs, yielding a total of 15 μs of trajectory data. The initial structures of the three MYC motifs were derived from the crystal structures “1NKP”, “8OTS” and “6G6K”, where “1NKP” and “8OTS” represent the MYC–MAX–DNA complex, and “6G6K” is the MYC–MAX complex. In each case, the MYC structure was isolated from its partners and extracted. The results from MD simulations are summarized in Figure 3 and include the average of the three longest trajectories. *C*_α_ RMSD values reported in Figure 3 were obtained taking a single crystal structure of MYC (1NKP) as the reference structure.

**Figure 3:**
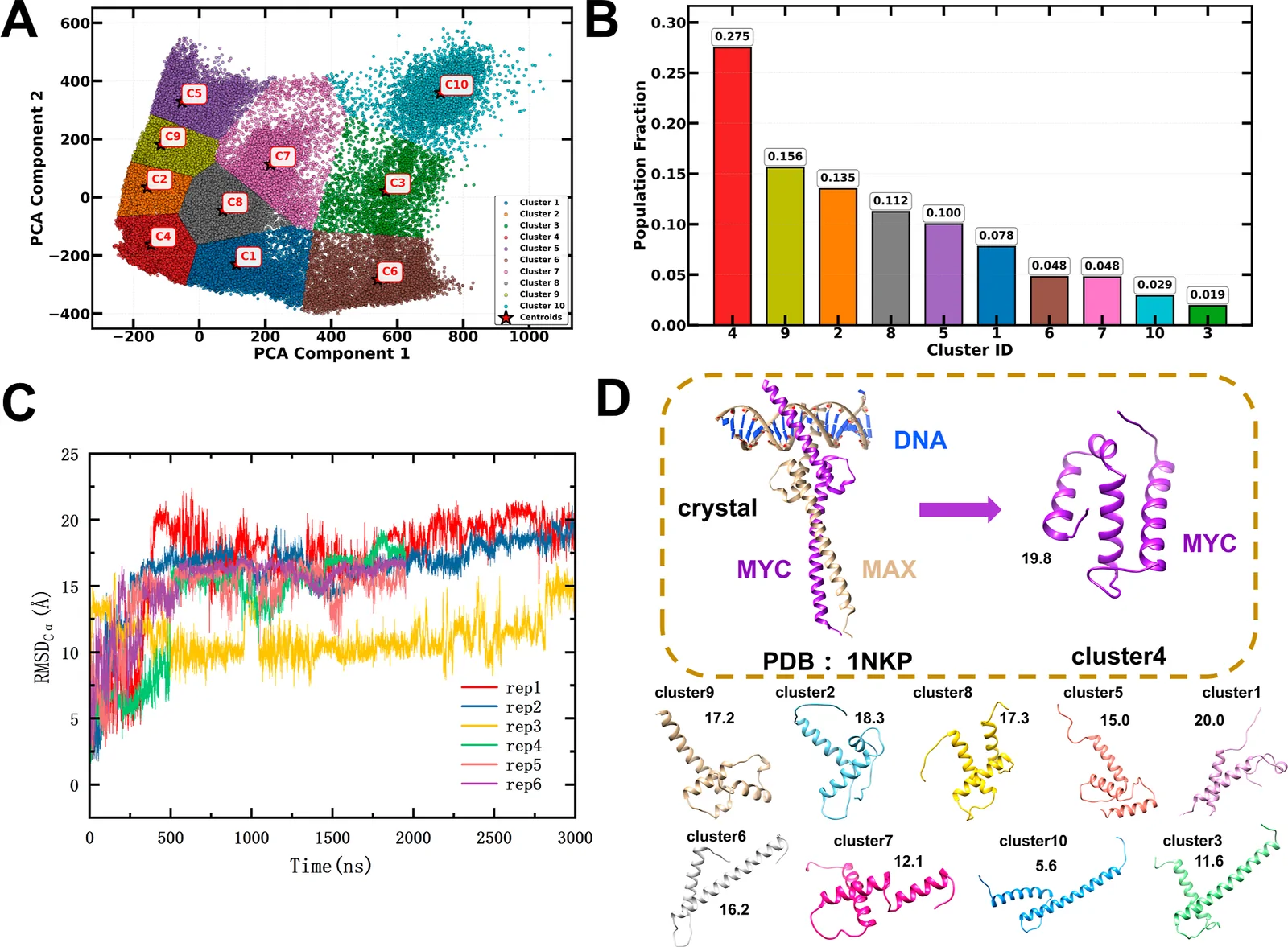
PCA clustering averaged from six independent MD runs, initialized with three different crystal structures “1NKP”, “6G6K” and “8OTS”. (A) Ten distinct clusters identified from PCA results, with the representative structure of each cluster (centroid) indicated by a red star; (B) cluster density distributions; (C) RMSD *C*_α_ profiles over the full simulation time of the six MD simulations, spanning a total of 3 μs (replicas 1–3) or 2 μs (replicas 4–6); (D) most stables structures (clusters 1–10) ordered by cluster density (top to bottom, left to right), starting with “cluster 4”. *C*_α_ RMSD of all clusters after superposition with the reference crystal structure of MYC (1NKP) are highlighted here by means of the small figures besides each cluster structure.

We can observe (Figure 3, panels A and B) that structure “4” is clearly the most populated of the ten detected ones, what indicates that such structure might correspond to the most stable one, holding for the largest part of the MD trajectories. Other structures such as those labeled as “9”, “2”, “8” and “5” have been recorded more than 10% of the total number of configurations analyzed. Interestingly, all cluster structures reported are qualitatively different of the CS of c-MYC published at PDB (1NKP). From supplementary RMSD data computed from the reference crystal structure 1NKP and the actual cluster structures, we can observe through the small figures included in Figure 3, that long-term RMSD values of clusters 4, 9, 2, 8, 5, and 6 range between 15 and 20 Å, while those of clusters 7, 10, and 3 range between 5.6 and 12.1 Å. This means that all clusters observed are significantly different of CS.

In addition, Figure 3C shows that the bHLH-Zip structure of MYC could not be maintained without MAX, exhibiting a relatively high RMSD. Significant changes in secondary structure and three-dimensional conformation (Figure 3D) occurred within approximately 1000 ns, subsequently maintaining a metastable state, as evidenced by the stabilization of the RMSD in 1000–3000 ns. However, within the conventional simulation time scale, the standard simulation struggles to sample the intrinsically disordered and highly flexible c-MYC monomer adequately. This is one of main reasons to conduct WTM simulation, since using bias forces allows us to assist MYC in overcoming the energy barrier and exploring a broad conformational pool. Structural cluster 4, which is representative of MYC’s conformations in these standard simulation trajectories and maintains relative stability, is a suitable choice for the initial structure in the WTM simulations, providing a basis derived from a cluster structure resembling physiological conditions to accelerate sampling. This strategy will allow us to determine whether the conformations identified by PCA, being totally different to the experimentally determined CS, correspond to thermodynamically stable or metastable states, since in general the most populated structures obtained from conventional unbiased MD trajectories will not necessarily coincide with the most stable conformations identified through enhanced-sampling methods such as WTM.

### Two-Dimensional Free Energy Landscapes of MYC

The two-dimensional (2D) free energy landscape of MYC is represented in Figure 4 for the two selected CV. We can observe that several main basins (such as A, B, ..., G) and their corresponding molecular conformations are well-defined in all cases. We have only indicated the basins which are clearly seen from the FES, assuming that even with well converged runs the clearest definition of the FES would require a huge amount of statistics out of the standard available ranges. A detailed study of the convergence of the WTM study is reported in the “Supporting Information”, Figures S1–S3.

**Figure 4:**
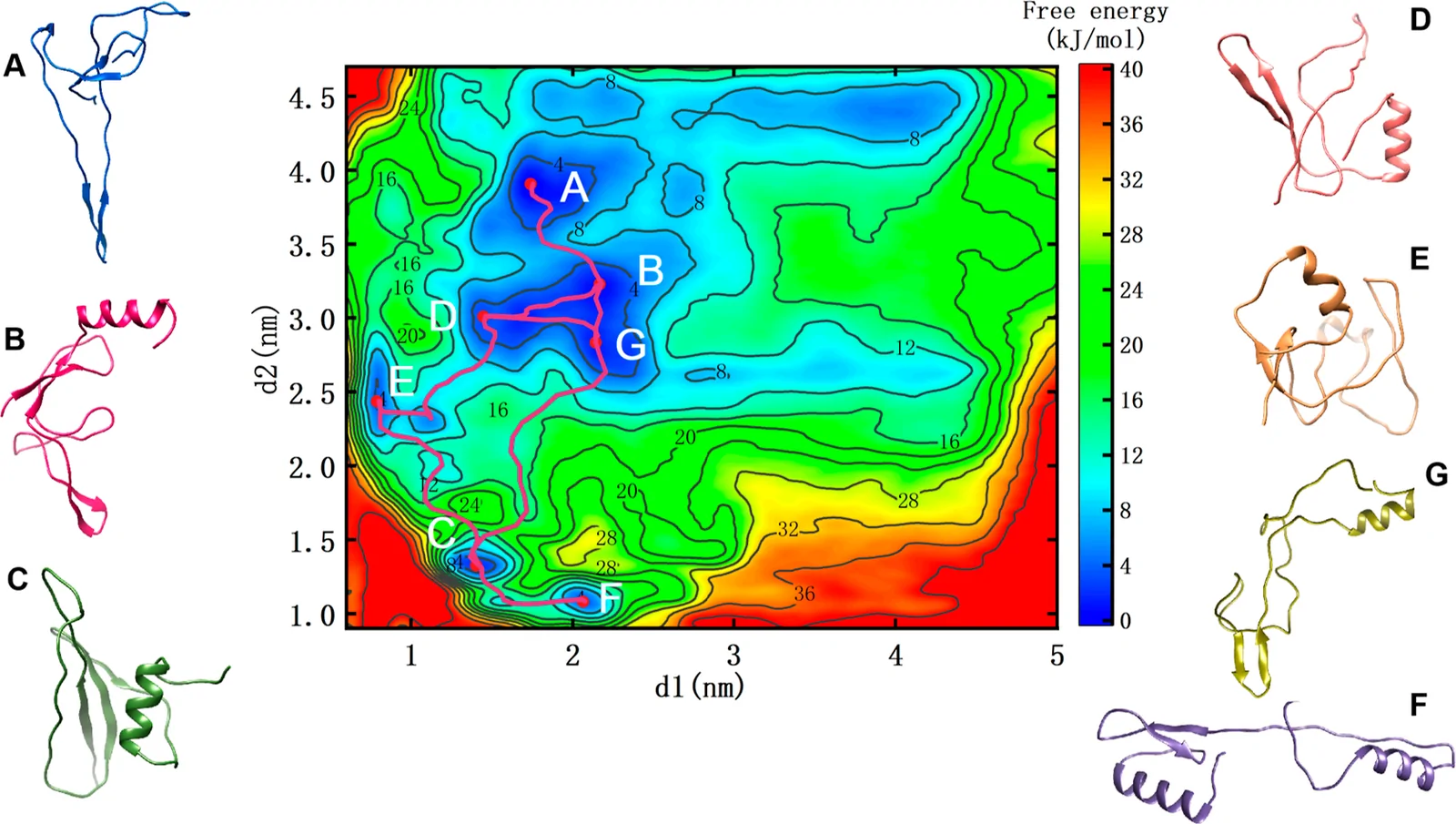
2D free energy landscape *F*(*d*_1_, *d*_2_) (kJ/mol) for MYC in solution. Basins “A”, “B”, “C”, “D”, “E”, “F” and “G” are the most thermodynamically stable states (minima) as they are explicitly indicated. The most visited conformations corresponding to the representative basins are listed on the two sides of the 2D free energy landscape. Minimum free energy paths are depicted in red lines.

As a general concept, the basins in the FES indicate the most probable configurations of the system in equilibrium. In fact, WTM is able to reveal those stable configurations in such a way that the free energy barriers that the system needs to overcome to shift between stable states can be estimated with a high degree of accuracy. In this work, we can distinguish several deep basins located around values of *d*_1_ between 0.79 and 2.17 nm and distances *d*_2_ between 1.08 and 3.90 nm. The coordinates of every selected basin (A–G) are reported in Table 2, together with their free energy values, where the minimum of all them (state A) has been set to zero, producing the corresponding rescaling of the remaining ones. Conversely, regions of higher free energy are less populated and correspond to MYC showing a growing disorder in its structure. An example of this effect can be observed in Figure S3D (basin H).

**Table 2:** Coordinates and free energies of the representative basins (minima) of MYC as reported in Figure 4, compared to the CS

free energy [kJ/mol]	basin	*d*_1_ [nm]	*d*_2_ [nm]
0.00	A	1.74	3.90
1.01	B	2.17	3.23
2.49	C	1.40	1.33
3.18	D	1.45	3.01
3.93	E	0.79	2.44
4.14	F	2.06	1.08
1.33	G	2.13	2.88
74.69	CS	7.26	2.30

As a general fact, the lowest free energy basins show values of *d*_1_ in a tight short-range around 1–2 nm, indicating that in all cases the distance between C- and N-terminals of MYC is quite short, suggesting “closed” and “packed” structures. Further, the wider range for *d*_2_ (about 1–4 nm) suggests a variety of different structures and shapes for the second α helix of MYC. Noticeably, the reported coordinate *d*_1_ of the CS is much larger than the ones of the stable basins, whereas *d*_2_ for CS falls right inside the same range of those of stable basins. This means that CS would fall in a high-energy region of the 2D FES (not reported in Figure 4).

Table 2 shows that basins A, B, C and G correspond to the regions of lowest free energy (0–2.5 kJ/mol) and may represent stable states that are structurally related to the native ensemble, but including relevant differences in loop orientations or side-chain packing. Otherwise, basins D–F are located in slightly higher-energy regions (between 3 and 5 kJ/mol), corresponding to less stable conformations, although these are basins that could be considered as containing potential drugging pockets as well. States with free energies beyond 10 kJ/mol have not been considered. Furthermore, we observe (Table 2) a substantial free energy difference of ∼75 kJ/mol between the most stable basin (A) and the experimentally resolved CS, which may be of great relevance for the development of drugs targeting MYC. It is worth noticing that during the entire WTM simulation, MYC did not recover its initial level of helicity, even though regions of the free energy landscape corresponding to the same CV values were revisited multiple times, suggesting that the crystal structure may not represent the most probable configuration in that region. For such a reason, designing inhibitors that target these low-energy conformations is potentially more promising in order to achieve stable binding between the MYC protein and the inhibitor. Conversely, templating on the crystal structure is likely to result in failed binding due to MYC’s spontaneous transition to lower-energy states. This provides a clear thermodynamic rationale for selecting structures A–G as target conformations. In passing, we should notice that a triplet formed by substates B, D and G is formed by three states located in a small region with similar free energy values and short transition half-life.

### Identification of Transition Paths and Transition States

Generally, basins in the FES indicate the most probable configurations of the system in equilibrium. As a matter of fact, WTM allows us to estimate the free energy barriers that the system needs to overcome to shift between stable states with a high degree of accuracy. Furthermore, maxima along each path give indications of the approximated location of TS between stable states. For instance, the TS between basins A and B is located around *d*_1_ ∼ 1.8 nm, *d*_2_ ∼ 3.6 nm with a free energy of about 7.2 kJ/mol. The full set of free energy profiles along the MFEP for each transition case are shown in Figure S5, including the estimated locations of local TS between stable basins. Regarding the stability and readiness of MYC to transit between the located basins, we report in Table 3 the free energy barriers for the basins of interest located from Figure 4, together with transition half-lives between these basins, estimated using the Eyring–Polanyi equation, eq 3.

**Table 3:** Free energy barriers (Δ*F*) between basins (kJ/mol) along the corresponding MFEP and their transition half-lives (ps), as predicted by the Nudged Elastic Band method^73^ and Eyring–Polanyi equation, with the transmission coefficient Set to 1 (see section “Methods”)^a^

transition between states	Δ*F* (kJ/mol)	transition half-life (ps)
A to B	7.21	1.76
B to A	6.21	1.19
B to D	2.48	**0.28**
D to B	0.31	**0.12**
B to G	1.42	**0.19**
G to B	1.10	**0.16**
D to G	0.72	**0.14**
G to D	2.57	**0.29**
D to E	6.70	1.45
E to D	4.69	**0.66**
E to C	16.21	57.77
C to E	17.65	101.02
C to F	13.04	16.92
F to C	11.40	8.93
C to G	13.01	16.68
G to C	8.69	3.12

a Transitions between stable states with half-lives shorter than 1 ps are shown in bold characters.

The energy barriers between low-energy basins and higher-energy regions (e.g., D to E, C to E, C to F and G to C) suggest that some large-scale rearrangements are thermodynamically unfavorable and would require to cross high activation barriers. Conversely, energy barriers between the lowest energy basins (e.g., A to B, B to D, B to G and D to G) are within a moderately narrow range of a few kJ/mol and indicate that some rearrangements thermodynamically favorable would require to cross low activation barriers. Half-life values along the MFEP between basins are consistent with the number of conformers sampled for each basin in the WTM trajectories and short half-lifes show faster kinetic transitions between stable states. In particular, structures of MYC corresponding to basins exhibiting very short transition half-lives (less than 1 ps) indicate that they are transient states, i.e. resonances between two states of the same physical structure and meaning such as, for instance, interconversion flips between chemical isomers. For instance, we have detected the transitions between B, D and G, with half-lives lower than 0.3 ps and energy barriers below the energy associated to thermal motion (2.6 kJ/mol at 310 K), what allow us to categorize the triplet of these 3 substates as the same physical state. The largest barriers (mainly involving transitions between C and E, F or G) indicate strong stability of these states. State A shows a much lower barrier but it only can transit to state B, so that we can recall that states A, C and B–D–G are the best initial candidates for the search of drugging pockets on their surface.

### One-Dimensional Free Energy Landscapes of MYC

The 2D free energy landscape obtained from WTM simulations (Figure 4) has been build using the two CV defined above. Nevertheless, it is possible to obtain one-dimensional (1D) profiles where free energy depends of only one CV, after the second CV has been integrated out. This sort of calculations will allow us to directly compare free energy barriers to experimental findings (see Jämbeck and Lyubartsev^59^), since information about binding modes in experimental solutes is usually not available. Then, it is possible to use experimental binding free energies Δ*G*_bind_ to get indirect leverage on the quality of the computed free energy barriers. We have computed the 1D profiles considering the integration procedure described by eq 1. The results reported in Figure 5 reveal a series of free energy barriers around the stable basins, most of them located very close to the lowest free energy state (A).

**Figure 5:**
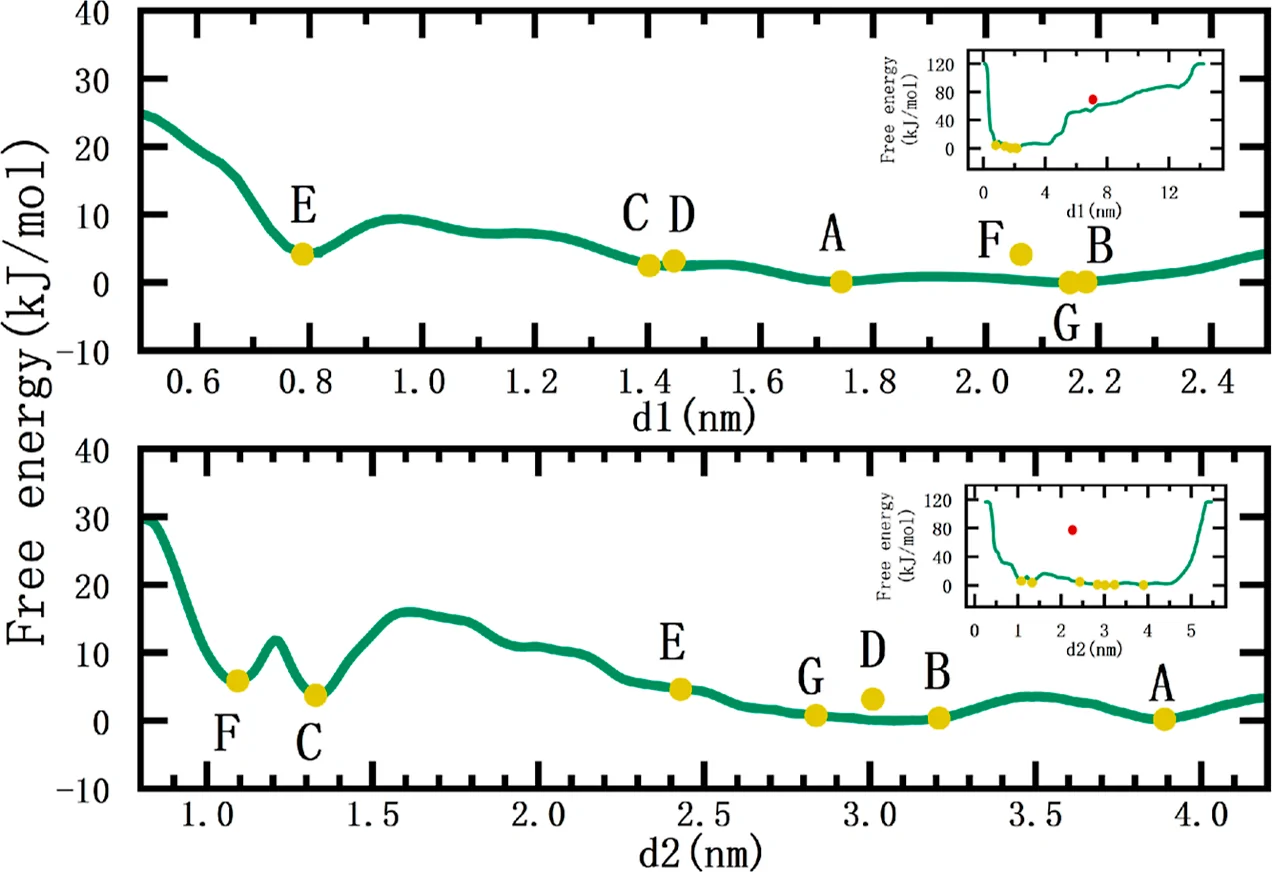
1D integrated free energy profiles for two CV. The top subfigure corresponds to the distance CV *d*_1_, whereas the bottom one corresponds to the CV *d*_2_. Corresponding insets show the full range of values considered for each CV. Basin regions marked with same labels (A–G) as in Figure 4. In order to directly compute the height of free energy barriers, absolute minima are set equal to zero. The yellow dots indicate the location of the seven lowest energy minima, whereas the red dots indicate the location of the CS.

The two free energy profiles *F*(*d*_1_) and *F*(*d*_2_) are quite smooth, showing very close values for the stable states (A–G). Most basins can be reached by crossing very low free energy barriers, as indicated in Table 3. The full free energy landscape spans *d*_1_ from 0 to 14.3 nm and *d*_2_ from 0 to 5.5 nm (insets in Figure 5). States A–G are concentrated in the low energy region of coordinate *d*_1_, reflecting the higher stability of compact structures. In contrast, states A–G are more widely distributed along the coordinate *d*_2_, providing a basis for MYC conformational diversity. All selected structures, excepting states E and F, have reached their lowest free energy for *d*_1_ or *d*_2_. Nevertheless, the CS is located at *d*_1_ = 7.26 nm, *d*_2_ = 2.30 nm, very far away from the most stable conformations, exhibiting a large difference of approximately 75 kJ/mol. We should notice that values of *d*_1_ of states F, B and G overlap, with B and G exhibiting lower energies. This results in point F for *d*_1_ of Figure 5 lying slightly above the free energy curve. A similar situation occurs for state D in the free energy projection on *d*_2_. Further, the energy barriers for F and C are primarily originated from the dependence on *d*_2_, with their free energy surface exhibiting a characteristic double-well. In summary, states A and B are the globally most stable structures (free energy difference of only 1.016 kJ/mol), whereas state C represents a moderately low free energy but structurally different of A and the triplet B–D–G.

Overall, the results for the free energy barriers reported here show many possible transitions between free energy basins involving free energies larger than the thermal energy at 310 K (∼2.6 kJ/mol). These barriers can be compared to other biological bottlenecks, such as those related to the RAS family of oncogenes, where barriers of ∼20 kJ/mol due to changes in specific distances and orientations were estimated for the activation of mutated RAS proteins by Fourier transform infrared spectroscopy^74^ and simulations^65^ or to the case of KRAS-G12D, when bound to a cell membrane, where angular barriers in the range 8–17 kJ/mol were estimated.^75^

### Location of Potential Drugging Pockets at the Surface of MYC

A possible way to determine the location of potential drugging pockets at the surface of the stable structures of MYC previously reported in sections “Two-Dimensional Free Energy Landscapes of MYC” and “One-Dimensional Free Energy Landscapes of MYC” is by plotting the charge densities at the MYC surface obtained from electrostatic calculations using the Poisson–Boltzmann equation. We have performed this calculation by means of the tool APBS (Adaptive Poisson–Boltzmann Solver^76^). The results are presented in Figure 6 and indicate the existence of clear spots where charge is neatly positive (red) and others where it is mostly negative (blue), whereas neutral regions are plotted in white.

**Figure 6:**
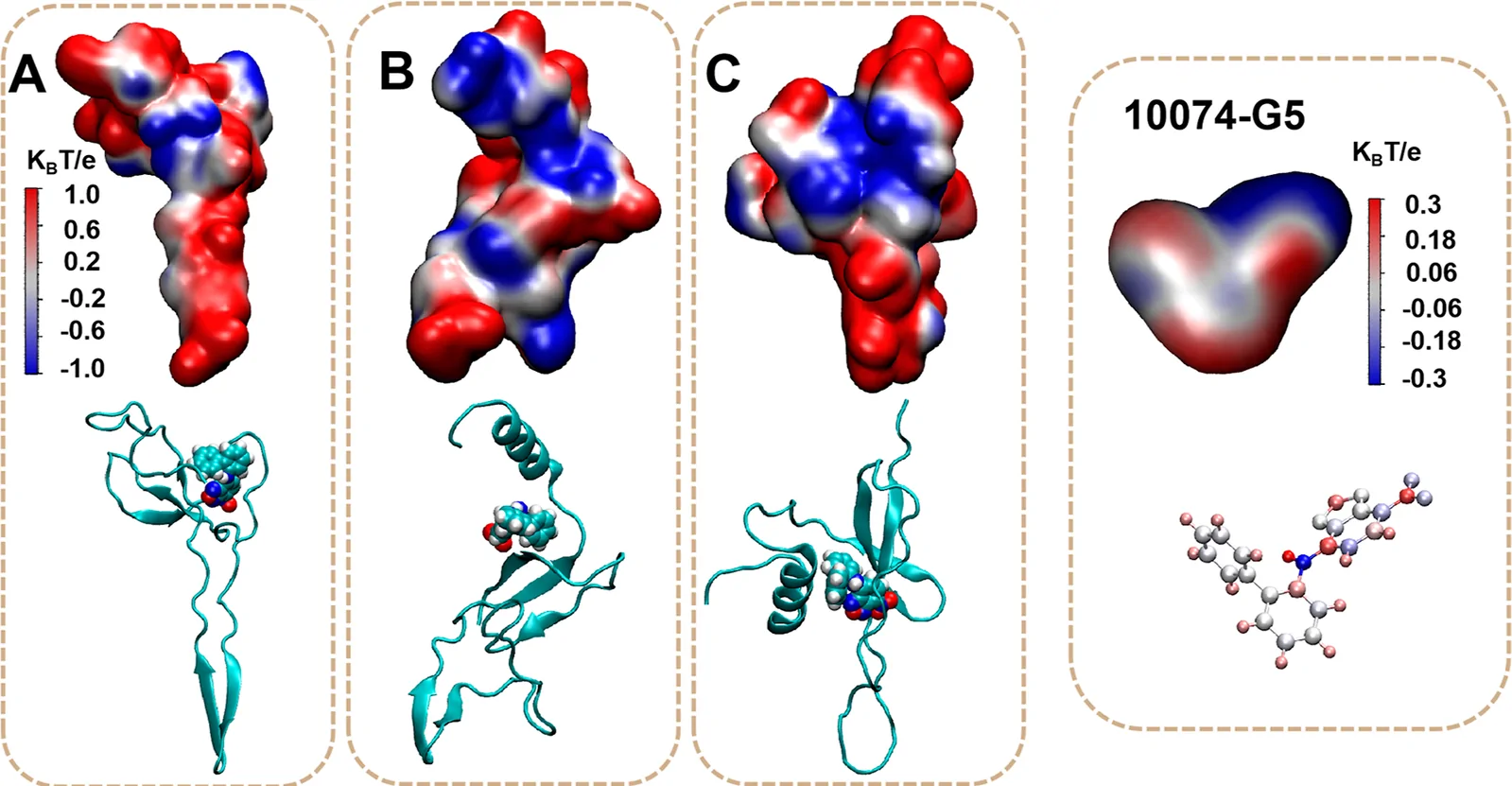
Electrostatic potential of selected MYC conformations in free energy basins A, B, and C (as reported in the 2D FES, Figure 4) in units of *k*_B_ *T*/*e*, where 1 *k*_B_ *T*/*e* ≈ 26.7 mV, highlighting positive (red) and negative (blue) side chains of each pocket. The most suitable drug-binding pocket of each state has been tested by allocating the small-molecule inhibitor 10074-G5,^20^ whose charge distribution and atomic structure are shown at the right side of the figure (For basins A, B and C, carbon atoms are depicted in cyan, hydrogens in white, nitrogens in dark blue and oxygens in red).

According to the selection of stable basins indicated above (namely minima A, B and C in Figure 4), we hypothesize that these states should be considered by their potential as promising therapeutic targets in MYC-associated diseases. Minima A, B and C not only correspond to the lowest energy in the potential landscape but also represent three relatively independent states with distinct conformations. Among these, B stands for three interconvertible subconformations: B, D and G. As it can be seen in Figure 6, there exist large pockets of these structures with electrostatic potential differences up to 2 *k*_B_ *T*/*e*, i.e. ∼53 mV. These negatively charged regions are complementary to the overall positive charge exhibited by the small molecule 10074-G5, indicating that they can form electrostatic interactions. In a similar fashion as it was described for MAX,^67^ we can assume that electrostatic interaction, say mediated by hydrogen bonds or salt bridges, might be a suitable mechanism for a small-molecule drug binding to MYC.

Our results suggest that the electrostatic binding of 10074-G5 to states A, B (resonance of triplet B–D–G), and C of MYC should be feasible given their different polar regions. In this way, we have conducted additional docking analyses between MYC and the 10074-G5 inhibitor, as predicted by Autodock Vina.^77,78^ The results of the docking analysis are reported in the Supporting Information (Table S1). We also included in Figure 7 the most favorable complex structures for states A, and C that agree well with our preliminary predictions.

**Figure 7:**
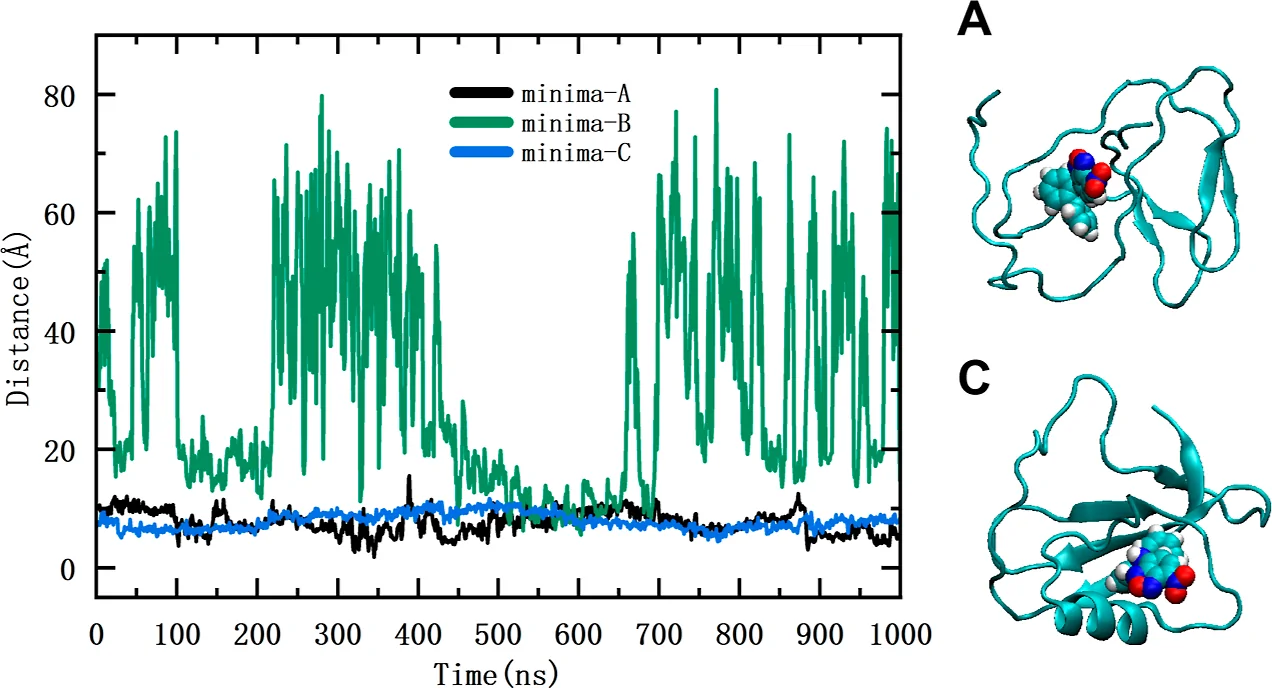
Time evolution of the distance between the center of mass of the MYC motif and the center of mass of the prototype drug 10074-G5 in the case of the three minima A, B, C (labeled as “minima-A, minima-B, minima-C”, as indicated in Figure 4). In the case of stable binding (configurations A, C) a snapshot of the final configuration is shown at right side. The inhibitor 10074-G5 is displayed in van der Waals representation and all figures were generated using VMD.

### Conformational Ensembles and the Mechanisms of Inhibition of Intrinsically Disordered Proteins

In order to study the stability of the complexes formed by the three states bound to the 10074-G5 inhibitor, we have run additional independent MD simulations and studied the distances between the center of mass of the 10074-G5 inhibitor and the center of mass of each MYC motif. The results of 1 μs runs are shown in Figure 7. State B tends to exhibit significant fluctuations, reflecting transient departures of the inhibitor from the center of mass of MYC followed by occasional returns on short time scales. These observations suggest that state B corresponds to a thermodynamically stable basin while remaining dynamically flexible, consistent with the short transition half-life observed between basin B and neighboring basins. In comparison, the binding of the 10074-G5 inhibitor into the drug pockets in states A and C of MYC are very stable, with distances located around 1 nm during the full period of simulation time. This result suggests that, for the particular inhibitor 10074-G5, at least two drugging pockets reported in the present work are able to host this drug during periods at the time scale of 1 μs. As an additional test, we have considered other higher-energy minima such as E and F, together with the D and G (B’s partners) and found that the distances between inhibitor-MYC center of mass are fluctuating significantly, indicating low stability as drugging pockets (see Figure S6). Our observation that 10074-G5 maintains steady binding within a specific pocket for about 1 μs must be interpreted within the context of recent advancements in intrinsically disordered proteins ensemble docking. As shown by Robustelli and co-workers,^25^ small molecules targeting disordered systems often undergo “dynamic shuttling” across multiple surface topologies. Furthermore, absolute binding free energy calculations of IDP ligands have highlighted that a single stable trajectory segment does not fully capture the macroscopic thermodynamic behavior due to the high entropic cost of binding. Therefore, the druggable pocket identified in our WTM simulations represents a highly populated, preorganized local structural landmark conformation that acts as an important focal interaction point within the broader, fuzzy structural ensemble.

To properly evaluate the structural insights from our WTM simulations, it is essential to distinguish the specific local sequence context of our target pocket from other frequently cited c-MYC small-molecule models. Crucially, prior computational work by Michel and Cuchillo^79,80^ focused exclusively on the behavior of the c-MYC C-terminal 402–412 segment, which corresponds to the cognate binding region of the rhodanine inhibitor 10058-F4. In contrast, our simulations characterize the independent and sequentially remote binding domain corresponding to the benzofurazan inhibitor 10074-G5, which maps to residues 363–381 within the N-terminus of the bHLHLZ motif. As established by biophysical mapping studies from Hammoudeh et al.,^23^ the 85-residue bHLHZip domain of monomeric c-MYC features at least three independent, nonoverlapping small-molecule binding sites that possess distinct local chemical and topological landscapes. The 10074-G5 pocket characterized in our work is framed by a highly specific cluster of basic residues (including 366, 367 and 378) adjacent to a local hydrophobic domain shaped by residues 375 and 381. This unique architecture accommodates the electron-rich nitrobenzofurazan ring and the bulkier biphenyl group of 10074-G5 through a structural mechanism entirely distinct from the purely hydrophobic collapse seen in the C-terminal 10058-F4 site. Therefore, our finding of a resilient, 1 μs persistent pocket interaction does not contradict the diffuse global energy landscape of c-MYC; rather, it provides the first detailed all-atom landscape map of a completely separate, charge-flanked metastable basin that behaves independently from the segments explored in earlier metadynamics studies.

## Concluding Remarks

This work reports a study of a motif c-MYC structures in solution. Determining the main conformations of this oncoprotein is crucial, as it forms a MYC–MAX dimer that binds to DNA inside the human cell nucleus. This complex plays a key role in dysregulating gene expression in about 70% of all known cancers. Given the lack of clinical strategies directly targeting MYC, identifying specific druggable pockets on its surface or interior is highly valuable for drug design. Here we present a detailed study of the free energy surface of c-MYC in solution to locate previously uncharacterized druggable pockets. To achieve this, we have combined MD-WTM simulations with auxiliary tools, including minimum free energy path and docking analyses for a 10074-G5 inhibitor. We observed that the unbound MYC motif exhibits a rich conformational ensemble, revealed by extensive MD and WTM simulations. MD simulations revealed seven dominant structural clusters of monomer, very different from those in the initial crystal structures isolated from the complex MYC–MAX–DNA obtained by X-ray and electron microscopy. These structures guided the selection of two distance-based collective variables for WTM enhanced sampling. The resulting FES revealed a broad conformational space featuring numerous low-energy basins corresponding to (meta)­stable states.

According to the findings reported in Figures 4 and 5, our FES shows stable states with low free energy barriers located at three different regions, as mapped by the two CVs. The most stable basin (A) has a free energy of ∼74.69 kJ/mol below the experimentally resolved crystal structure, corroborating that the latter is a low-populated state in solution. The next two low-energy basins (B and C) have been also selected to explore potential drugging pockets. These pockets represent promising candidates for effective inhibitor targeting, moving beyond the long-studied MYC–MAX crystal structures. Finally, electrostatic potential maps of states A and C reveal polar pockets that showed good affinity and dynamical stability in a range of 1 μs when bound to the small-molecule MYC inhibitor 10074-G5, highlighting potential targets for future drug development.

## Supplementary Material



## Data Availability

The raw data and analysis scripts that support the findings of this study can be found in the corresponding repository of Zenodo: 10.5281/zenodo.21514201. The repository contains: input files for WTM simulations, coordinates of meta- and stable MYC structures (related to the use of the PLUMED plugin), scripts for PCA calculations, raw block analysis data, minimum free energy pathways between different basins, the optimized structure of 10074-G5, along with docking results for 10074-G5 and the most favorable MYC-inhibitor complex structures predicted through docking simulations. In addition, topology, parameter and MD input files are also provided.

## Notes

Convergence of well-tempered metadynamics simulations, including convergence of free energy profiles and block size averages of estimated errors; free energy profiles along minimum free energy paths indicating the location of transition states for each transition between basins; details on 10074-G5 and MYC docking simulations, including list of docking scores; dynamical stability of selected potential drugging pockets (PDF)
