# Supplementary material for: Drugging Pockets at the Surface of c‑MYC Oncoproteins Revealed by Well-Tempered Metadynamics
Source: J Chem Inf Model. 2026 Oct 22;66(21):14025–37. doi: 10.1021/acs.jcim.6c02197 (PMC13753514; doi:10.1021/acs.jcim.6c02197)
Supplement: Supplementary file 1 [file ci6c02197_si_001.pdf]

Supporting Information for "Drugging pockets at the surface of c-MYC oncoproteins revealed by well-tempered metadynamics"

Yanhong Ge,[†] Huixia Lu,[*,†,‡] and Jordi Marti[*,†]

†*Department of Physics, Universitat Politècnica de Catalunya-Barcelona Tech (UPC), Campus Nord UPC, 08034, Barcelona, Spain*

‡*Institut de Ciència de Materials de Barcelona (ICMAB-CSIC), Carrer dels Til·lers, 08193, Cerdanyola del Vallès, Spain*

E-mail: huixia.lu@upc.edu; jordi.marti@upc.edu

We will focus this complementary set of data on: (1) Convergence of WTM simulations; (2) Location of TS for each transition between basins; (3) List of docking scores and (4) Stability of specific potential drugging pockets.

Convergence of WTD simulations

To ensure the reliability of the WTM simulations reported in the present work we can use several criteria. Three of the most used are: (1) As a basic initial test, the time evolution of the fluctuations of the hills of the biased potential along the full trajectory; (2) The time cumulative average of 1D free energy profiles and (3) The so-called block analysis of the average error along free energy profiles as a function of the block length.

The evolution of the hills is presented in Figure S1. We observe an overall decreasing behavior, eventually including high spikes during the transient part of the simulation (0-2000

ns). The height of the biased potential decreases accordingly along the simulation run, so that a quasi-flat profile is already seen around 4000 ns, including a few low-height spikes. Afterwards, a few high spikes are observed when the simulation reaches higher-energy regions (between 6000 and 7500 ns), but such regions are of small importance and the fluctuations are not physically relevant, as we will see below. We should remark that this is an approximate but not yet conclusive indication of the convergence of the WTM runs.

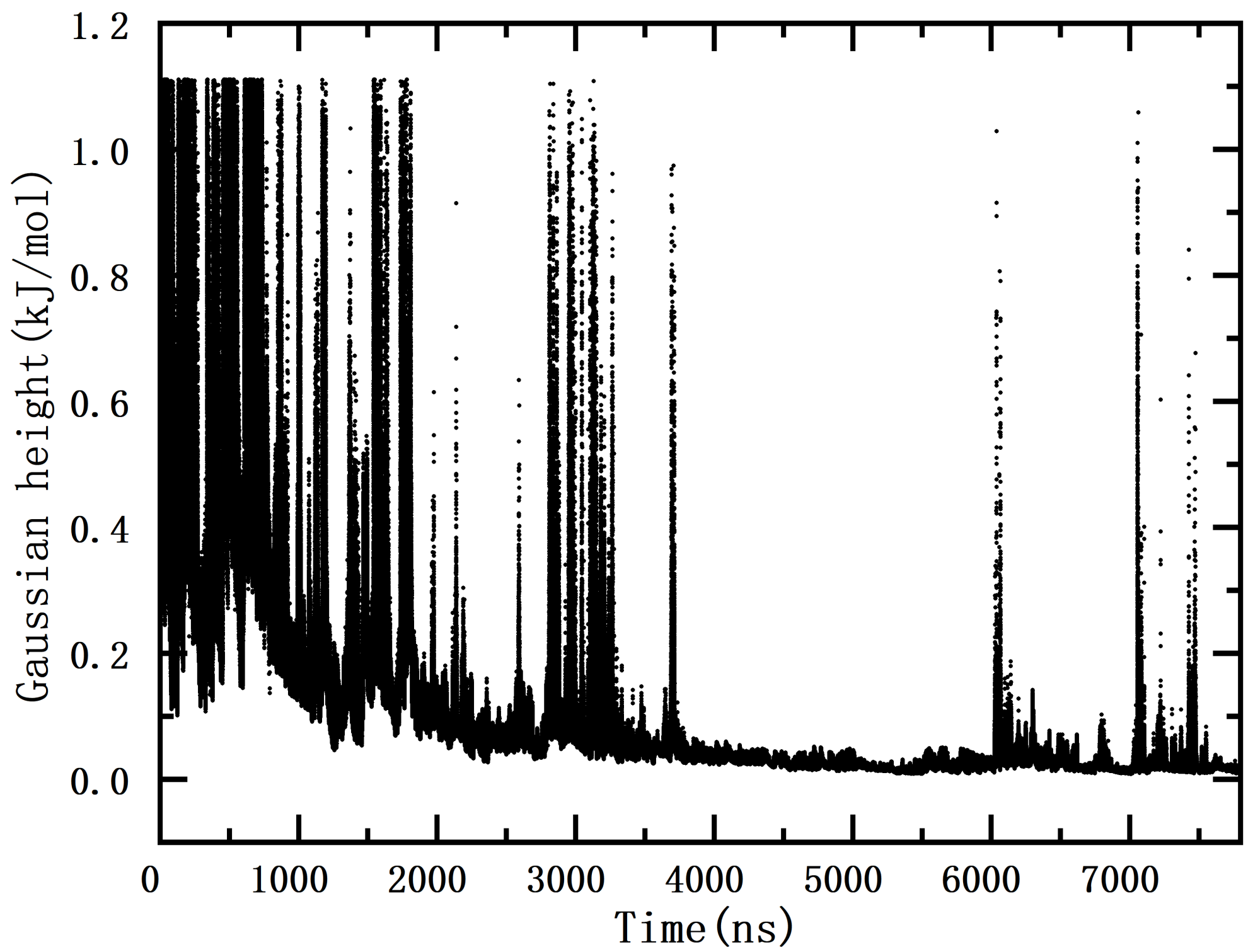

Figure S1: Time-evolution of WTM's hills

Further, from the results of Fig. S2 we can see that after long cumulative time lengths, the differences between a profile and the one immediately before are quite small (less than 20 kJ/mol), leading us to fully converged free energies for the two CV considered in the present study.

Further, whereas in Fig. S1 a few high spikes indeed appear between 6000 and 7500 ns, we can correlate them with the results from panels A and B of Fig. S3. The CV regions explored during this period are d1 ∼(2.5, 5.5) and d2 ∼(3.5, 5.5), can be located in the

full "map" of panel Fig. S3C (marked with a black line). These are not core low-energy regions, indicating that the trajectory's final stage explored thermodynamically relatively active structures. Fig. S3C shows that the region explored between 6000 and 7800 ns also exhibited a local energy minimum, with the structure being noticeably more loosely packed and thermodynamically more active, as shown in Fig. S3D. The high structural activity of MYC poses a significant challenge for simulations, but this does not preclude its use. The disordered nature of the MYC structure results in a greater structural diversity, causing our simulation to converge after running for 7800 ns which is far exceeding normal simulation duration.

Finally, for the sake of reaching full safety in the convergence tests, we report the size of the average error in the free energy profiles calculated from WTM simulations as a function of the block size in Fig. S4. These estimated errors are computed as the size of the average error in the free energy profiles calculated from two different sets of WTM simulations as a function of the block size. The initial 25% of all metadynamics trajectories were discarded. This is the so-called block analysis of the average error along free energy profiles as a function of the block length. This method consists on dividing the data (N values) in n chunks or blocks of equal size s ($n \times s = N$). Then, the method will compute the mean value for each block, the mean of these block means, and the standard error for the latter (the mean of means).[1–3] As expected, the errors increase with the block length until they reach a plateau for the two CVs. The average errors tend to stabilize around 1.2 kJ/mol for *d1* and 1 kJ/mol for *d2*, indicating consistent behavior over extended simulation times. Both criteria indicate that the WTM simulations performed in this study have converged. For this calculation, scripts provided by the PLUMED project[2,3] have been employed.

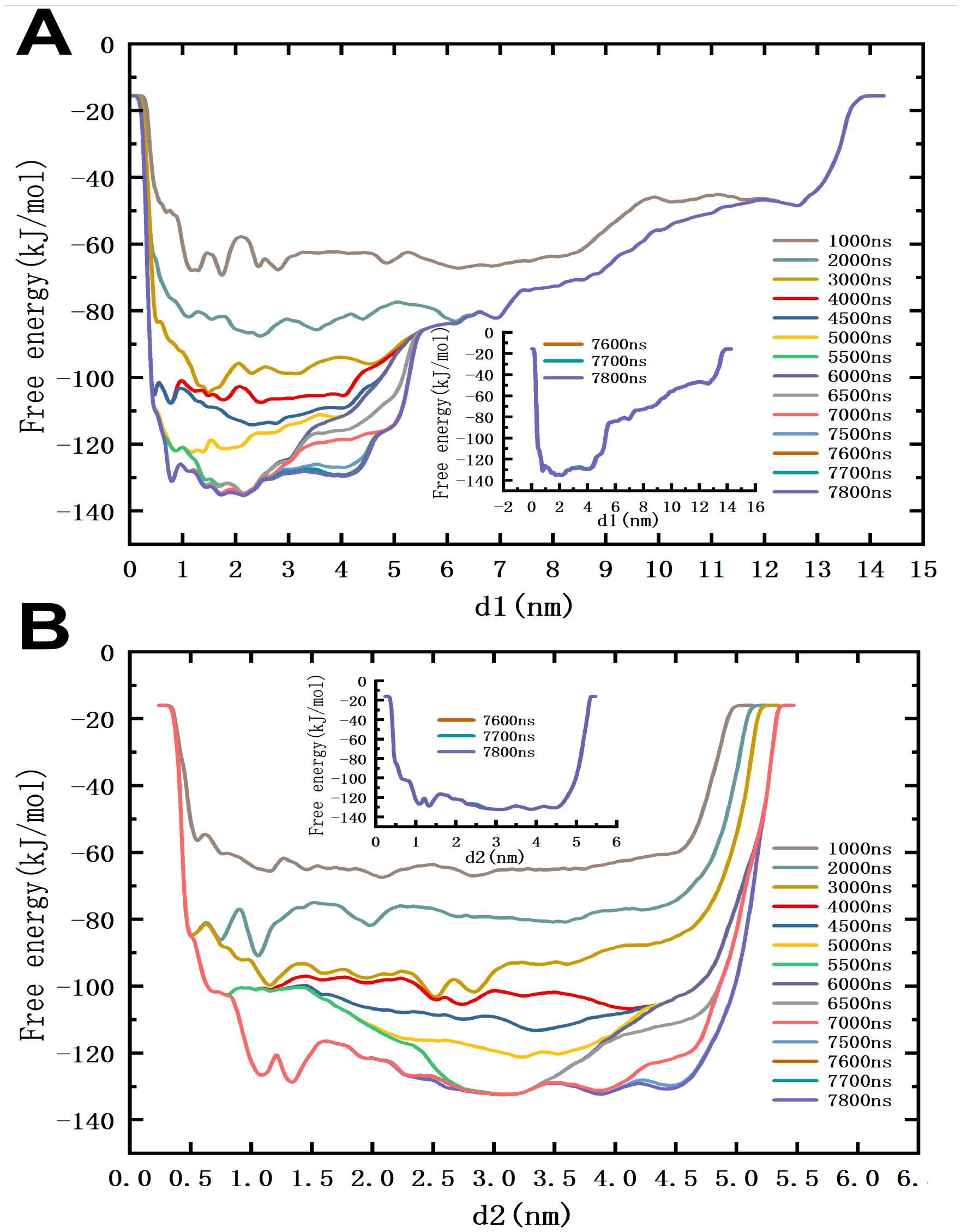

Figure S2: Convergence of one-dimensional free energies at different simulation times from 1000 to 7800 ns. (A) Free energy as a function of *d1*, (B) free energy as a function of *d2*.

Free energy profiles along the minimum free energy paths

In the main text, we reported the minimum free energy paths (MFEP) connecting different free energy basins as identified by the Nudged Elastic Band method[4] (Fig. 4 of main text). Although the free energy values can be identified from that figure, in order to complete the set of data, we show here the free energy profiles explicitly evaluated over these paths (Fig. S5). We indicate the TS found along these paths.

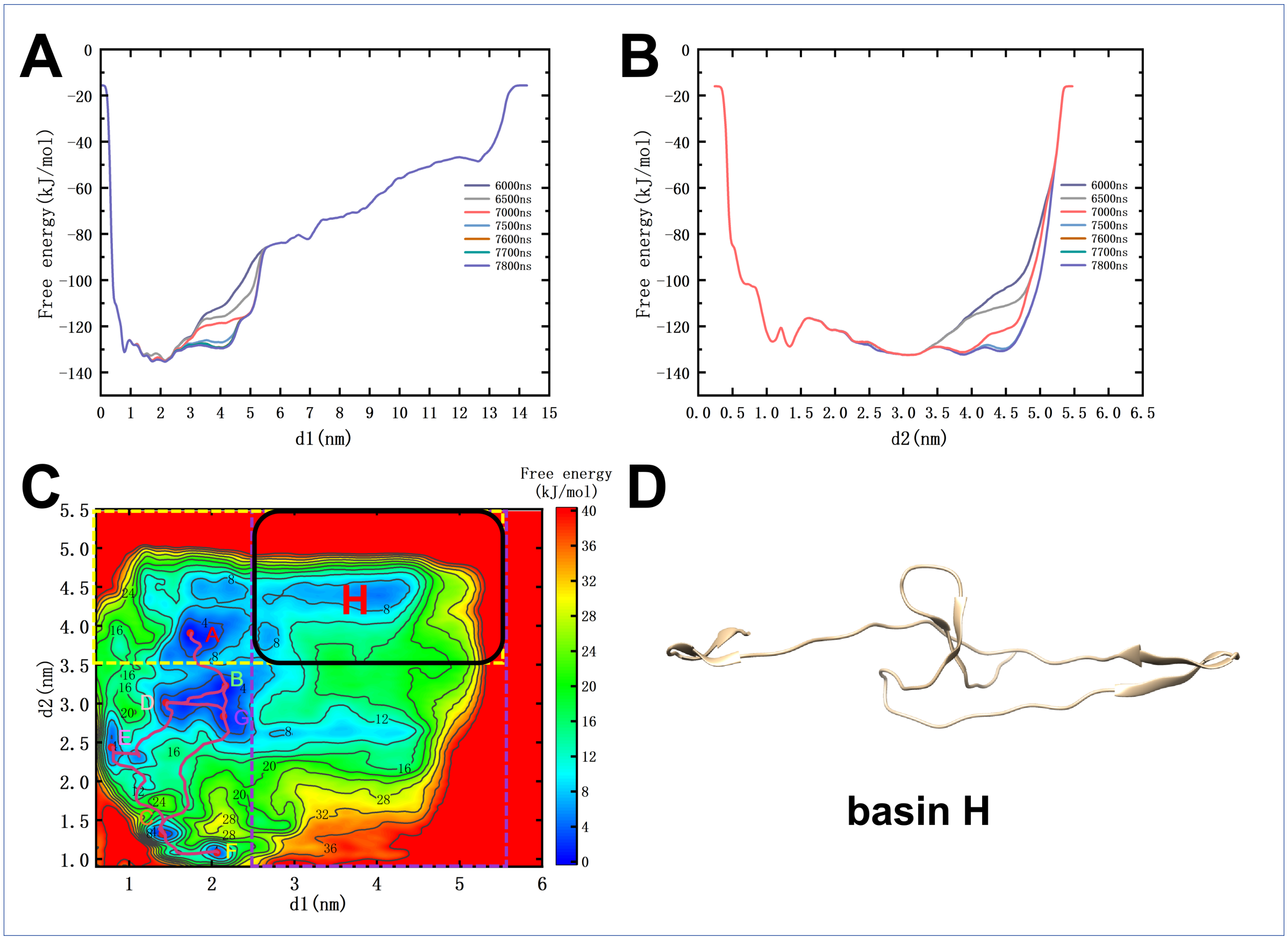

Figure S3: Convergence of one-dimensional free energies in the range 6000-7800 ns. (A) Free energy as a function of *d1*, (B) free energy as a function of *d2*. (C) Detailed region sampled by WTM during the interval over the 6000-7800 ns. (D) Typical MYC configuration at the region (basin) H.

10074-G5 and MYC docking simulations

We used AutoDock Vina[5,6] to perform docking simulations for basins A–G with the small-molecule inhibitor 10074-G5, in order to predict their binding modes and affinities. The results of the docking scores between MYC and the 10074-G5 inhibitor are listed in Table S1. AutoDock Vina was employed to dock 10074-G5 with these pockets. The complex structure with the best docking score is shown in Figure 6 of the main text. The inherent scoring function of AutoDock Vina yields the predicted binding free energy, evaluating the binding affinity between ligand and target. Decreasing values indicate stronger predicted binding affinity. Guided by established thresholds in drug screening, molecules with scores below

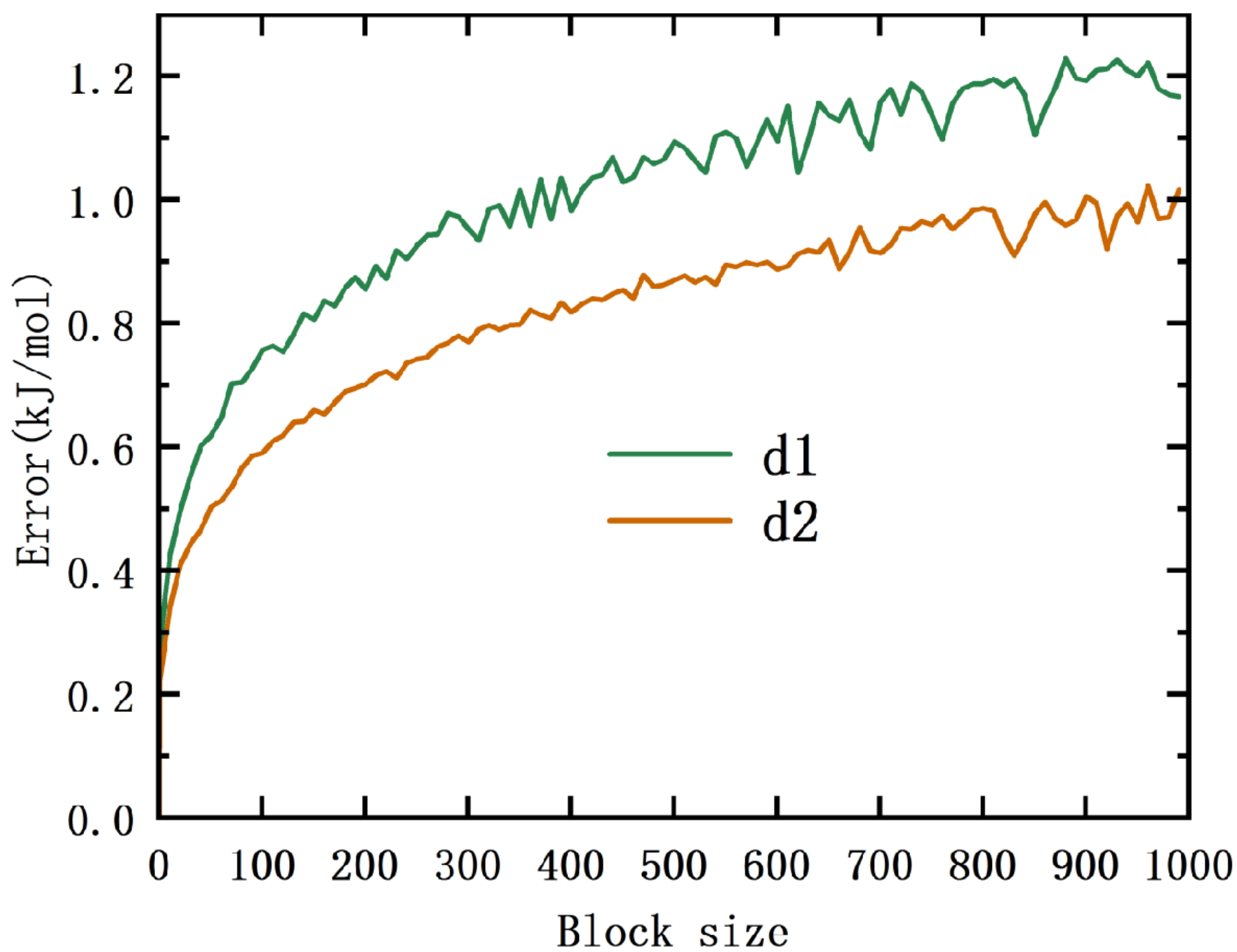

Figure S4: Block-size averages, for the two CV employed in this work.

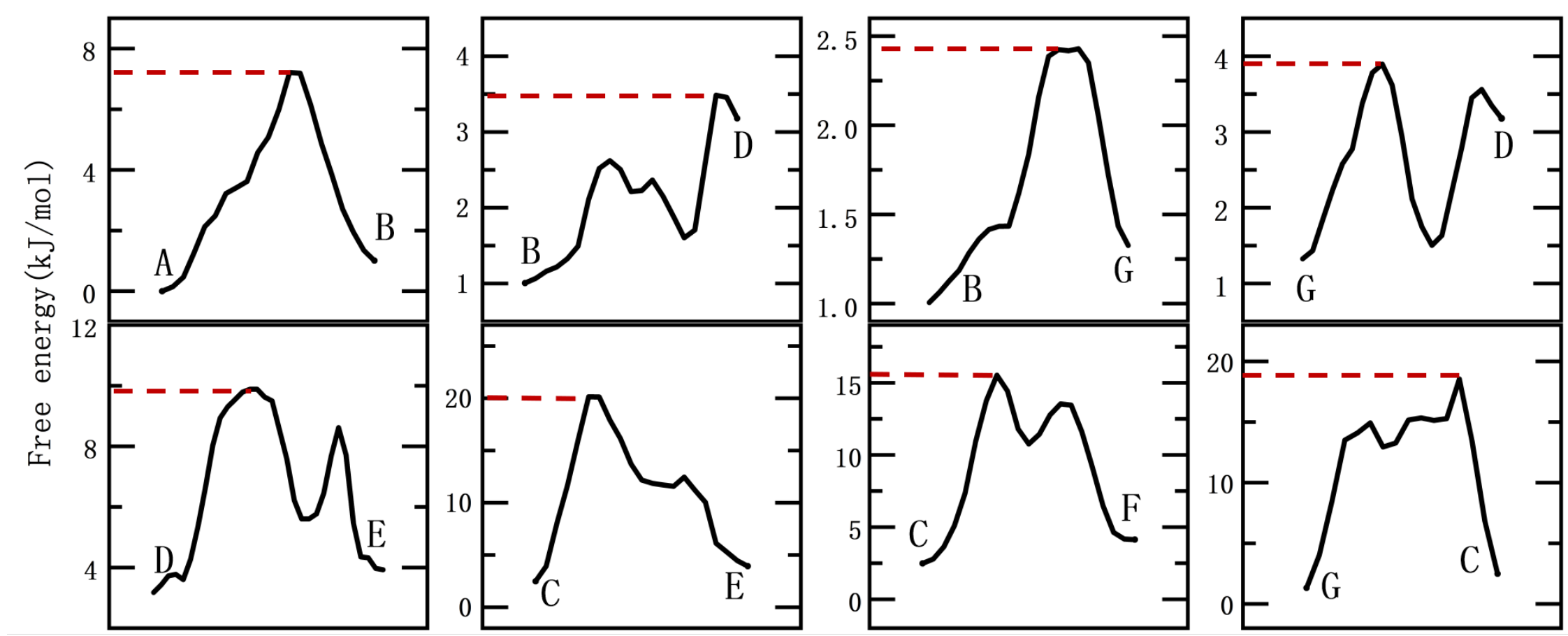

Figure S5: Free energy profiles evaluated over the MFEP shown in Fig. 4 of the main text. TS are indicated with a dashed line.

−7.0 kcal/mol are generally considered to possess strong binding potential. All states A-G qualified under this criterion.

Table S1: Docking scores

State	Docking score (kcal/mol)
A	-7.8
B	-7.1
C	-8.9
D	-7.5
E	-8.1
F	-7.4
G	-7.9

Stability of specific potential drugging pockets

In a similar fashion as the results presented in Fig. 6 of main text, we report here (Fig. S6) a stability study of pockets D, E, F, and G showing a bad stability performance overall.

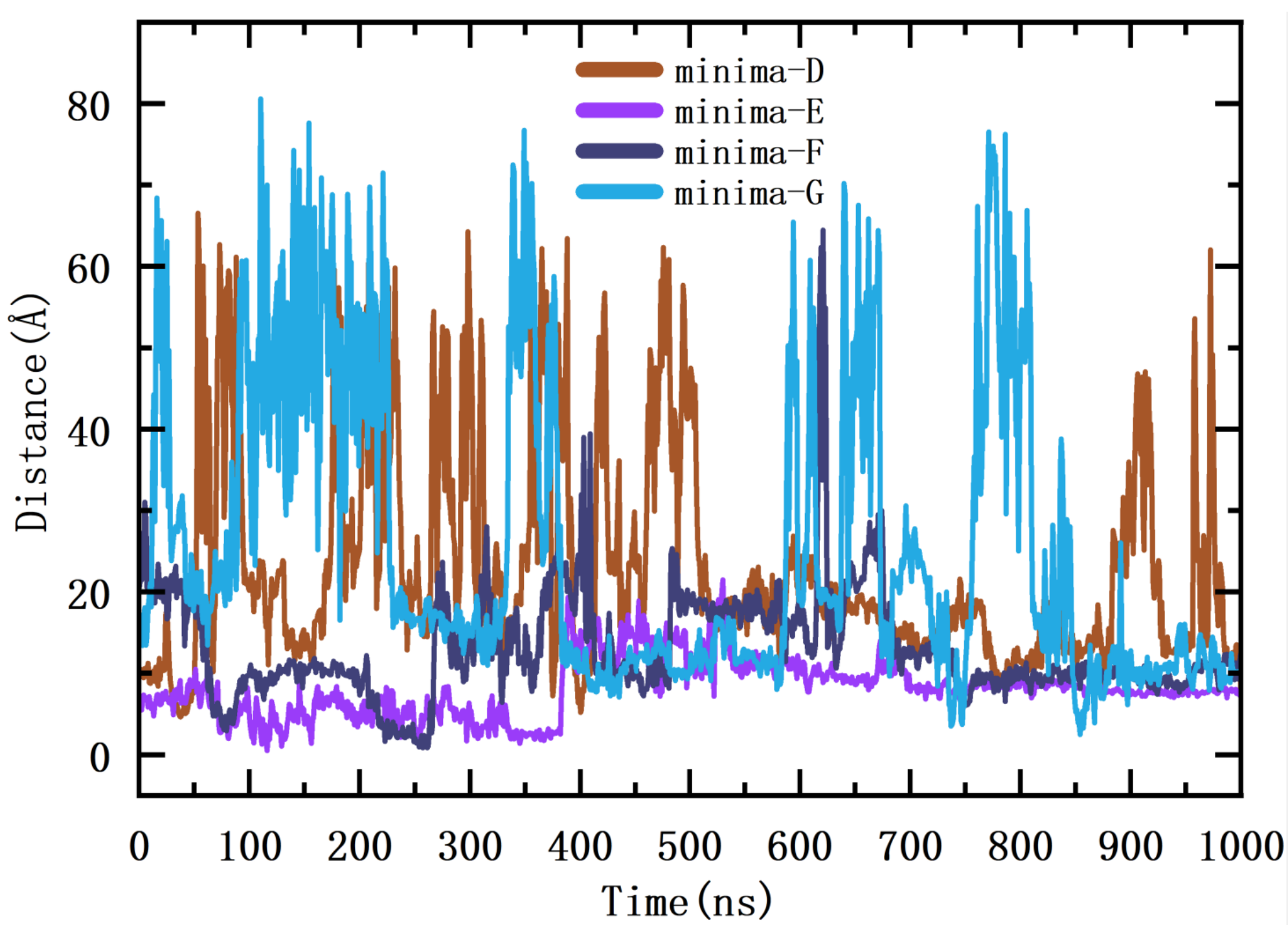

Figure S6: Time evolution of the distance between the center of mass of the MYC motif and the center of mass of the prototype drug 10074-G5 in the case of the minima D, E, F and G (as indicated in Fig. 4 of main text).

References

(1) Flyvbjerg, H.; Petersen, H. G. Error estimates on averages of correlated data. *The Journal of Chemical Physics* **1989**, *91*, 461.

(2) Bonomi, M.; Branduardi, D.; Bussi, G.; Camilloni, C.; Provasi, D.; Raiteri, P.; Donadio, D.; Marinelli, F.; Pietrucci, F.; Broglia, R. A.; others PLUMED: A portable plugin for free-energy calculations with molecular dynamics. *Computer Physics Communications* **2009**, *180*, 1961–1972.

(3) Tribello, G. A.; Bonomi, M.; Branduardi, D.; Camilloni, C.; Bussi, G. n. PLUMED 2: New feathers for an old bird. *Computer physics communications* **2014**, *185*, 604–613.

(4) Henkelman, G.; Jónsson, H. Improved tangent estimate in the nudged elastic band method for finding minimum energy paths and saddle points. *The Journal of chemical physics* **2000**, *113*, 9978–9985.

(5) Trott, O.; Olson, A. J. AutoDock Vina: improving the speed and accuracy of docking with a new scoring function, efficient optimization, and multithreading. *Journal of computational chemistry* **2010**, *31*, 455–461.

(6) Eberhardt, J.; Santos-Martins, D.; Tillack, A. F.; Forli, S. AutoDock Vina 1.2. 0: new docking methods, expanded force field, and python bindings. *Journal of chemical information and modeling* **2021**, *61*, 3891–3898.
